# Cytotoxicity of targeted PLGA nanoparticles: a systematic review†

**DOI:** 10.1039/d1ra00074h

**Published:** 2021-03-03

**Authors:** Hock Ing Chiu, Nozlena Abdul Samad, Lizhen Fang, Vuanghao Lim

**Affiliations:** Integrative Medicine Cluster, Advanced Medical and Dental Institute, Universiti Sains Malaysia Bertam 13200 Kepala Batas Penang Malaysia vlim@usm.my +604-5622427; School of Pharmacy, Xinxiang Medical University Xinxiang Henan 453003 People's Republic of China

## Abstract

Recent advances in nanotechnology have contributed tremendously to the development and revolutionizing of drug delivery systems in the field of nanomedicine. In particular, targeting nanoparticles based on biodegradable poly(lactic-*co*-glycolic acid) (PLGA) polymers have gained much interest. However, PLGA nanoparticles remain of concern for their effectiveness against cancer cells and their toxicity to normal cells. The aim of this systematic review is to identify a promising targeting PLGA nanoformulation based on the comparison study of their cytotoxicity potency in different cell lines. A literature search was conducted through the databases of Google Scholar, PubMed, ScienceDirect, Scopus and SpringerLink. The sources studied were published between 2009 and 2019, and a variety of keywords were utilized. In total, 81 manuscripts that met the inclusion and exclusion criteria were selected for analysis based on their cytotoxicity, size, zeta potential, year of publication, type of ligand, active compounds and cell line used. The half maximal inhibitory concentration (IC_50_) for cytotoxicity was the main measurement in this data extraction, and the SI units were standardized to μg mL^−1^ for a better view of comparison. This systematic review also identified that cytotoxicity potency was inversely proportional to nanoparticle size. The PLGA nanoparticles predominantly exhibited a size of less than 300 nm and absolute zeta potential ∼20 mV. In conclusion, more comprehensive and critical appraisals of pharmacokinetic, pharmacokinetic, toxicokinetic, *in vivo* and *in vitro* tests are required for the investigation of the full value of targeting PLGA nanoparticles for cancer treatment.

## Abstract 2

Targeted drug delivery systems using PLGA as a nanocarrier for anti-cancer agents to improve efficacy and reduce toxicity in normal cells.

## Graphical abstract

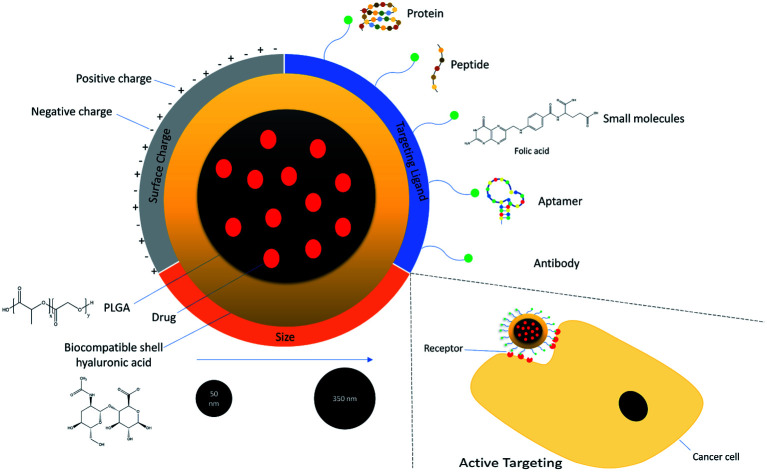

## 1. Introduction

Recent advances in nanotechnology have contributed tremendously to the development and revolutionizing of the drug delivery system in the nanomedicine field. The application of nanoparticles has long been recognized as a controlled release formulation for delivering a therapeutic agent to a specific targeted site. Nanoparticles provide a high therapeutic effect against cancers, which has earned them remarkable research interest among researchers. Nanoparticles offer a highly efficient targeted therapy compared to traditional cancer therapies. This targeted therapy can be done easily on nanoparticles using an ideal targeting ligand. Thus, targeting nanoparticles based on biodegradable polymers have gained much interest for treating cancer cells with minimal systemic side effects.^1,2^

**Fig. 1:**
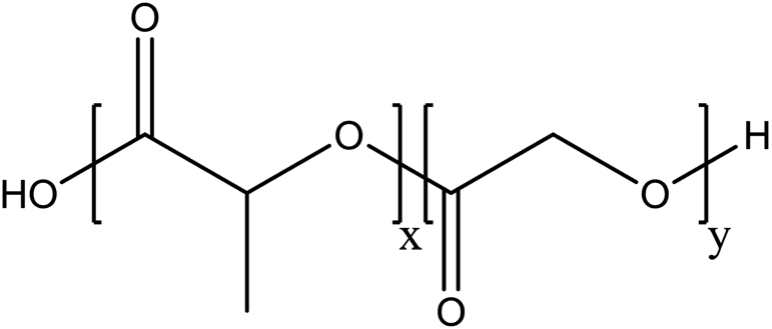
Chemical structure of PLGA.

Poly(lactic-*co*-glycolic acid) (PLGA) is a biodegradable polymer that has attractive properties as a nanocarrier for cancer therapy. PLGA is a hydrophobic copolymer and mainly composed of two monomers: lactic acid and glycolic acid (Fig. 1). PLGA is approved by the European Medicine Agency and the US Food and Drug Administration (FDA) as an ideal material for designing a drug delivery system due to its biocompatibility and biodegradability. PLGA is widely adapted for preparing nanoparticles encapsulating hydrophilic and hydrophobic anti-therapeutic agents.^3,4^ PLGA offers an enhanced permeability and retention (EPR) effect, sustained and controlled drug delivery for cancer therapy, enhanced accumulation of drugs in tumor vasculature and targeted delivery by surface conjugation with targeting ligands.^5^

Active and passive mechanisms are often practiced when targeting cancer cells using nanoparticles. Passive targeting is the application of polymeric nanoparticles owing to their size, shape and surface charge enabling them to be accumulated predominantly in the microenvironment of cancer cells *via* the EPR effect. This EPR effect is amplified based on the exclusive presence of leaky vasculature and impaired lymphatic drainage in tumors. Active targeting is the attachment or grafting of targeting/biorecognizable ligands on nanoparticles to target specific receptors/biomarkers that are overexpressed in cancer cells, excluding normal cells.^6^ Since targeting ligands are highly selective towards overexpressed receptors in cancer cells, in which can result in enhanced cellular uptake of nanoparticles as well as excluding harm to normal cells. Both passive and targeting mechanisms are considered a gold standard in designing a drug delivery system.

Recently, the use of PLGA nanoparticles for cancer therapy has received great interest due to the advantages offered and approval by the FDA. However, the safeness of PLGA nanoparticles containing cytotoxic therapeutic agent remains a huge concern. The anti-cancer drugs lack targeting specificity to cancer cells and could induce potent cytotoxic effects against both normal and cancer cells. In addition, the cellular concentration of the drugs is relatively low in cancer cells due to the low efficiency of non-targeting PLGA nanoparticles in delivering the drugs to the site of action. The cancer cell targeting of PLGA nanoparticles, enhanced cellular uptake of the drugs and low toxicity to normal cells are the most important criteria or measurements for chemotherapy. Therefore, it is vital for researchers to design a PLGA nanocarrier that, in addition to being biocompatible, biodegradable and cost-effective, can specifically release drugs at the target site with reduced systemic effects.

Hence, this systematic review is focused on a comparison study of the cytotoxicity potency of the targeting PLGA nanoparticles on the basis of published *in vitro* assessment findings from 2009 to 2019 in order to assess the PLGA nanoparticles with the ideal targeting ligands for specific cell lines – preferentially based on their IC_50_ cytotoxicity potency – and to correlate the size and zeta potential of nanoparticles with cytotoxicity potency.

**Fig. 2:**
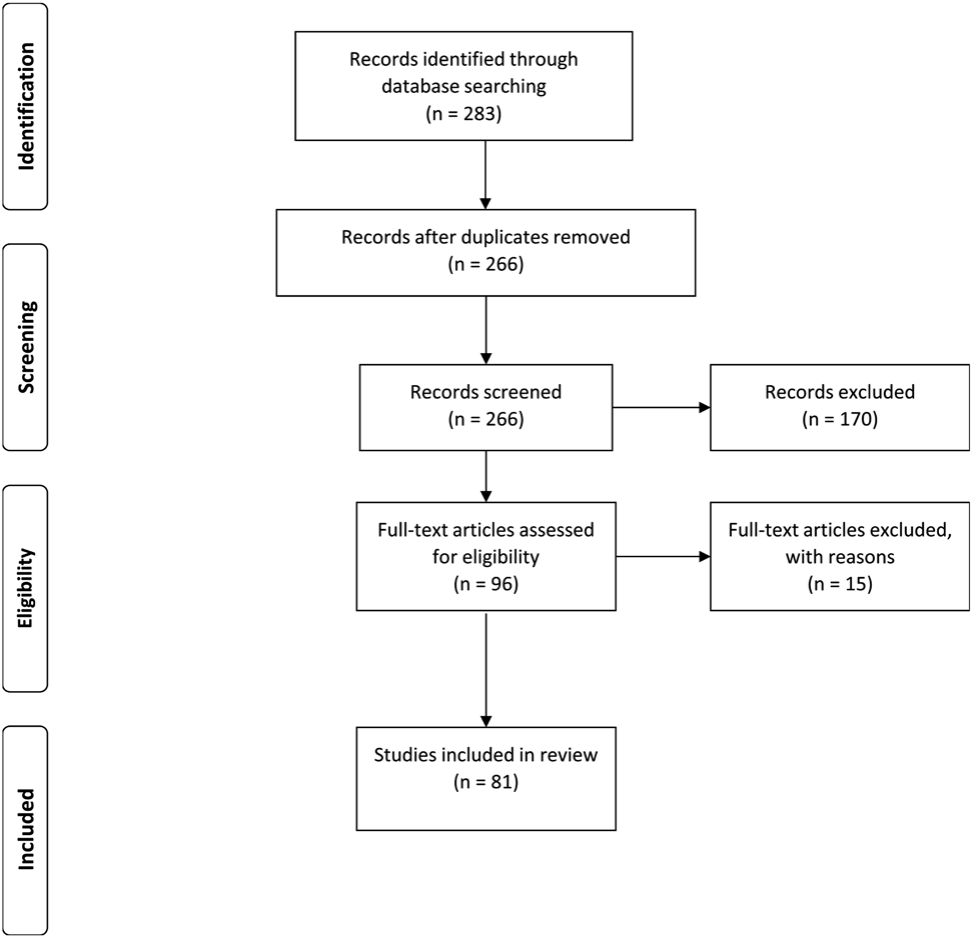
Flowchart of the selection of studies, using PRISMA guidelines.

The databases of Google Scholar, PubMed, ScienceDirect, Scopus and SpringerLink were searched for literature published between 2009 and 2019. Different combinations of keywords – including PLGA nanoparticles, cytotoxicity, targeting ligands and anti-cancer – were used for the literature search (Fig. 2). The methodology for the study was based on the Preferred Reporting Items for Systematic Review and Meta-Analysis Protocols (PRISMA-P) 2015.^7–9^ The inclusion criteria for our study were: (1) PLGA nanoparticles with different types of targeting ligands; (2) PLGA with nanoencapsulated active compounds and exhibited cytotoxic effects on normal and cancer cells; (3) PLGA with co-encapsulation of outer or inner polymers; (4) studies published between 2009 and 2019 (including in-press articles). Studies with the following criteria were omitted (exclusion criteria): (1) PLGA microparticles; (2) chemical conjugation of PLGA with other polymers to form nanoparticles; (2) all *in vivo*, *ex vivo*, *in silico*, clinical studies, and review articles; (4) studies without available cytotoxicity data; (5) articles that were not published in English. Based on the inclusion and exclusion criteria, the articles that fulfilled the requirements were selected for analysis. Screening of the articles was conducted by two independent reviewers. The data extraction involved analysis of the selected articles based on the types of cells, IC_50_ for cytotoxicity, year of publication, treatment duration, types of active compounds used, types of targeting ligands, types of studies (*in vitro*) and the size and zeta potential of the nanoparticles. The data were described and presented in a table. The IC_50_ for cytotoxicity was the main measurement in this data extraction. The SI units were standardized to μg mL^−1^ for a better view of comparison. The IC_50_ is the dose required to inhibit 50% of the cell viability. Based on the availability of the data of IC_50_, size and zeta potential of the nanoparticles, PLGA nanoparticles with the active targeting properties were selected for the purpose of studying the correlation between the particle size/zeta potential of PLGA nanoparticles and cytotoxicity potency.

## 2. Cytotoxicity of PLGA nanoformulations

The databases of Google Scholar, PubMed, ScienceDirect, Scopus and SpringerLink were searched. These produced 113, 32, 113, 20 and 5 articles, respectively. From all the databases, a total of 266 articles were retrieved after 17 duplicates were removed. Following this, 170 articles that were not compliant with the inclusion criteria were identified and excluded from the study. The 96 articles left were thoroughly assessed according to the exclusion criteria defined in Introduction. After critical assessment, 15 articles were omitted due to the methodology and cytotoxicity data being insufficiently described. Hence, 81 studies have been integrated into the qualitative synthesis involving assessment of the data of *in vitro* studies.

### 2.1 *In vitro* studies

The data of the cytotoxicity of PLGA nanoparticles conjugated with particular targeting ligands that deliver specific active compounds against different types of cells – such as brain, breast, lung, colon, stomach, gastric, liver, ovary, cervix, prostate, uterus, pancreas, skin, umbilical vein endothelial, esophagus, bladder, head, neck and kidney cells – are shown in Table 1. From the table, it can be seen that PLGA nanoparticles are time/dose-dependent on cytotoxicity.

**Table 1:** The cytotoxicity of PLGA nanoparticles on different types of cells

Type of cell line		IC_50_	Cell viability assay	Size (nm)	Zeta potential (mV)	Active compound	Exposure duration	Response relationship	Targeting ligand	Ref.	Year
Brain	U87	1.03 μg mL^−1^	MTT	∼150	−18.1 ± 0.5	Doxorubicin and paclitaxel	48 h	Dose-dependent	Transferrin	10	2013
		0.13 μg mL^−1^					96 h	Time-dependent			
		Not available (NA)	SRB	∼190	+38.6 ± 4.1	Doxorubicin and EGFR siRNA	48 h	Dose-dependent	Angiopep-2	34	2015
	U87MG	NA	XTT	181.9 ± 4.5	+40.212 ± 2.844	Etoposide	6, 12, 24 and 48 h	Time-dependent	Lactoferrin and FA	35	2015
	C6	NA	MTT	182.8 ± 3.78	−11.72 ± 2.27	Paclitaxel	24, 48 and 72 h	Time-dependent	Transferrin	36	2009
		7.28 μg mL^−1^	WST-1	138.5 ± 7.0	−43.03 ± 0.38	Paclitaxel	24 h	Dose-dependent	Vitamin E TPGS	37	2019
	GI-1	NA	MTT	85.5	−26.5 ± 2.1	Paclitaxel	24, 48 and 72 h	Time-dependent	AS1411	38	2012
		NA	MTT	∼200	NA	Paclitaxel	24, 48 and 72 h	Time-dependent	AS1411	39	2012
	U251	0.185 μg mL^−1^	CCK-8	137.3 ± 8.6	−17.63 ± 3.10	Doxorubicin	48 h	Dose-dependent	Chondroitin sulphate	40	2019
	SH-SY5Y	NA	MTT	257.10 ± 22.39	−5.51 ± 0.73	Doxorubicin	26 h	Dose-dependent	Rabies virus-derived peptide	41	2017
	DKMG/EGFRvIII	NA	MTT	251 ± 3	−2.9 ± 1.2	Curcumin	24 h	Dose-dependent	Anti-EGFRvIII monoclonal antibody	42	2018
Breast	D2F2	>0.114 μg mL^−1^	MTS	124.2 ± 21.2	+12 ± 7	PE38KDEL	48 h	Dose-dependent	Fab' fragments of a humanized anti-HER2 monoclonal antibody (rhu-MAbHER2)	43	2009
	D2F2/E2	0.00078 ± 0.00024 μg mL^−1^									
	SKBR-3	0.00497 ± 0.000176 μg mL^−1^	MTS	124.2 ± 21.2	+12 ± 7	PE38KDEL	48 h	Dose-dependent	Fab' fragments of a humanized anti-HER2 monoclonal antibody (rhu-MAbHER2)	43	2009
		0.00031 to 0.00049 μg mL^−1^	MTS	170.3 ± 7.6	−18.9 ± 1.5	Aromatase inhibitor, 7α-(4′-amino)phenylthio-1,4-androstadiene-3,17-dione (7α-APTADD)	24 h	Dose-dependent	Transferrin	11	2010
		NA	MTS	180 ± 1	−1.0 ± 0.1	Paclitaxel	24 h	Dose-dependent	Herceptin	44	2013
	MCF-7	6.71 μg mL^−1^	MTT	110	−32	DDP prodrug (c,c, t-[Pt(NH_3_)_2_Cl_2_(O_2_CCH_2_CH_2_CH_2_CH_2_CH_3_)_2_])	24 h	Dose-dependent	Folate	45	2012
		NA	Alamar blue	218	−27 ± 3	Paclitaxel	120 h	Dose-dependent	AS1411	46	2013
		NA	MTS	268.3 ± 23.4	+24.21 ± 2.31	Epirubicin	24, 48, 72, 96 and 120 h	Time-dependent	Biotinylated chitosan	47	2016
		∼0.043 μg mL^−1^	MTS	∼180	−22	Paclitaxel and combretastatin A4	24 h	Dose-dependent	Cyclo-(Arg–Gly–Asp–d-Phe–Lys)	48	2010
		NA	MTT	85.5	−26.5 ± 2.1	Paclitaxel	24, 48 and 72 h	Time-dependent	AS1411	38	2012
		NA	MTT	222.7	+10.2	Epirubicin	72 h	Dose-dependent	5TR1 DNA aptamer (Apt)	49	2017
		NA	MTT	245.8 ± 7.8	−8.57 ± 0.6	Gold nanorods and doxorubicin	28 h	Dose-dependent	Human serum albumin	50	2019
		0.02611 μg mL^−1^	MTT	274 ± 1.6	−13.8 ± 5.1	Rapamycin	120 h	Dose-dependent	EGFR antibody	51	2009
		0.42 ± 0.15 μg mL^−1^	MTT	141 ± 58.41	−2.61 ± 1.37	Epirubicin	24 h	Dose-dependent	Trastuzumab	52	2019
		1.84 ± 1.30 μg mL^−1^	MTT	153.41 ± 4.78	+49.1 ± 1.2	Salinomycin and paclitaxel	24 h	Dose-dependent	Hyaluronic acid	53	2016
		0.053 ± 0.0065 μg mL^−1^ (48 h), 0.039 ± 0.0052 μg mL^−1^ (72 h)	MTT	184.6 ± 4.1	−28.6 ± 2.4	Docetaxel, silibinin and SPIONs	48 and 72 h	Time-dependent	LHRH decapeptide hormone	54	2016
		NA	MTT	210.4 ± 10.14	+9.7 ± 0.2	Antimir-21 and epirubicin	72 h	Dose-dependent	MUC1 Apt	55	2018
		8.12 μg mL^−1^	MTT	151.7 ± 4.9	−1.00 ± 0.11	Paclitaxel	48 h	Dose-dependent	Glutamate	56	2016
	HER2-negative MCF7	NA	MTT	268.4 ± 6.6	+11.80 ± 0.75	Paclitaxel	24, 48 and 72 h	Time-dependent	Herceptin	57	2016
	HER2-positive BT474	NA	MTT	268.4 ± 6.6	+11.80 ± 0.75	Paclitaxel	24, 48 and 72 h	Time-dependent	Herceptin	57	2016
	BT-20	2.73 ± 0.28 μg mL^−1^	MTT	141 ± 58.41	−2.61 ± 1.37	Epirubicin	24 h	Dose-dependent	Trastuzumab	52	2019
	MDA-MB-453	3.11 ± 0.19 μg mL^−1^	MTT	141 ± 58.41	−2.61 ± 1.37	Epirubicin	24 h	Dose-dependent	Trastuzumab	52	2019
	MDA-MB-231	NA	MTS	286.20 ± 4.35	−15.97 ± 1.28	Docetaxel	24, 48, 72 h	Time-dependent	Hyaluronic acid ceramide	58	2014
		4 μg mL^−1^	MTT	150 ± 13.32	−21.57 ± 2.93	Quercetin	24 h	Dose-dependent	Transferrin	59	2018
		0.048 ± 0.004 μg mL^−1^	WST-1	91.2 ± 8.1	−60.7 ± 1.0	Doxorubicin and irinotecan	240 h	Dose-dependent	Hyaluronic acid	60	2015
	MBA-MD-231/ADR	NA	MTT	198 ± 12	−19.6 ± 1.5	Doxorubicin	48 h	Dose-dependent	FA	61	2016
	4T1 mammary epithelial carcinoma cells	NA	MTT	235.5 ± 71.30	NA	Curcumin and bortezomib	24 h	Dose-dependent	Alendronate	62	2012
		0.37 μg mL^−1^	MTT	151.7 ± 4.9	−1.00 ± 0.11	Paclitaxel	48 h	Dose-dependent	Glutamate	56	2016
	MCF-7/ADR	4.6 μg mL^−1^	MTT	∼210	+27	Doxorubicin	48 h	Dose-dependent	Low-molecular weight protamine	63	2014
		NA	MTT	245.8 ± 7.8	−8.57 ± 0.6	Gold nanorods and doxorubicin	28 h	Dose-dependent	Human serum albumin	50	2019
	JC	0.034 ± 0.00085 μg mL^−1^	MTS	240 ± 1	−19 ± 5	Paclitaxel and tariquidar	96 h	Dose-dependent	Biotin	64	2009
		NA	MTS	200–250	−12.1 ± 0.3	Paclitaxel and P-gp-targeted siRNA	24 h	Dose-dependent	Biotin	65	2010
	T47D	NA	MTT	210 ± 10	−13	Paclitaxel	48 h	Dose-dependent	Human serum albumin	66	2015
Lung	A549/T	0.78 μg mL^−1^	MTT	∼210	+27	Doxorubicin	48 h	Dose-dependent	Low-molecular weight protamine	63	2014
	A549	0.34 μg mL^−1^	MTT	∼210	+27	Doxorubicin	48 h	Dose-dependent	Low-molecular weight protamine	63	2014
		30 μg mL^−1^	MTS	286 ± 10.3	−45 ± 3.2	Doxorubicin	20 h	Dose-dependent	Cyclo-(1,12)-PenITDGEATDSGC (cLABL) peptide	67	2009
		NA	MTT	301 ± 10	−37.4 ± 1.4	Doxorubicin	24 h	Dose-dependent	LFC131 peptide	68	2014
		0.00330 μg mL^−1^	SRB	108 ± 12.5	−21.32 ± 1.91	Doxorubicin	48 h	Dose-dependent	Transferrin	69	2015
		NA	MTS	200	58.07	Chlorin e6	24 h	Dose-dependent	Hyaluronic acid	70	2017
	A549-Luc	4.12 μg mL^−1^	MTT	58	−29.4	Docetaxel	48 h	Dose-dependent	Hyaluronic acid	71	2017
		0.91 μg mL^−1^	MTT	154	−22.7	Docetaxel	48 h	Dose-dependent	Hyaluronic acid	72	2016
	A549-luc-C8	NA	MTT	80	−50	Paclitaxel palmitate	48 h	Dose-dependent	Cetuximab	73	2013
	TFR positive A549	0.01 μg mL^−1^	CCK	96–156	NA	Hypocrellin A	8 h	Dose-dependent	Transferrin	74	2017
	H1299, A549 and H1975	A549 (∼0.011 μg mL^−1^), H1299 (∼0.024 μg mL^−1^), H1975 (∼0.0017 μg mL^−1^)	Trypan blue	217 ± 13.54	+29.5 ± 4.04	Paclitaxel	24 and 48 h	Time-dependent	RGD peptide	12	2017
Colon	Caco-2	1.019 ± 0.233 μg mL^−1^	MTT	330 ± 3	−3.9 ± 0.3	Paclitaxel	24 h	Dose-dependent	Wheat germ agglutinin	13	2010
		0.087 ± 0.020 μg mL^−1^					72 h	Time-dependent			
	CCD-18Co	1.007 ± 0.121 μg mL^−1^					24 h	Dose-dependent			
		0.137 ± 0.027 μg mL^−1^					72 h	Time-dependent			
	Ht-29	0.061 ± 0.021 μg mL^−1^	MTT	330 ± 3	−3.9 ± 0.3	Paclitaxel	24 h	Dose-dependent	Wheat germ agglutinin	13	2010
		0.028 ± 0.008 μg mL^−1^	MTT	330 ± 3	−3.9 ± 0.3	Paclitaxel	72 h	Time-dependent	Wheat germ agglutinin	13	2010
		NA	MTT	90 ± 1.9	−36.3 ± 4.2	Curcumin	2 + 48 h	Dose-dependent	Ribonucleic acid (RNA) Apts against epithelial cell adhesion molecule (EpCAM)	75	2014
	CT26	NA	MTT	245.8 ± 7.8	−8.57 ± 0.6	Gold nanorods and doxorubicin	28 h	Dose-dependent	Human serum albumin	50	2019
	C26	NA	MTT	210.4 ± 10.14	+9.7 ± 0.2	Antimir-21 and epirubicin	72 h	Dose-dependent	MUC1 Apt	55	2018
		3.67 μg mL^−1^	MTT	102.3 ± 2.9	−13.2 ± 2.4	10-Hydroxy camptothecin (HCPT)	48 h	Dose-dependent	Chondroitin sulphate	76	2019
	HCT116	NA	MTT	150 ± 12	+0.2 ± 0.12	Camptothecin	72 h	Dose-dependent	Conatumumab (AMG 655)	77	2011
Stomach	MKN74, MKN74-CD44v6+ and GP202	NA	MTT	293 ± 15	−20.0 ± 0.4	Paclitaxel	24 and 48 h	Time-dependent	Human anti-human CD44v6 Fab, AbD15179	14	2018
Gastric	HGC27	0.05 μg mL^−1^	MTT	406.6 ± 65.5	−25.0 ± 2.0	SN38 (7-ethyl-10-hydroxycamptothecin)	48 h	Dose-dependent	AHNP	39	2016
	MKN28	NA	MTT	∼200	NA	Pheophorbide a	2 h	Dose-dependent	Folate	78	2018
Liver	HepG2	0.78 μg mL^−1^	MTT	138 ± 3.12	NA	Epirubicin	24 h	Dose-dependent	LFC131 peptide	79	2016
		0.38 μg mL^−1^					48 h	Time-dependent			
		5.4 ± 0.21 μg mL^−1^	MTT	204	−5.6	Docetaxel	72 h	Dose-dependent	Human serum albumin	80	2013
		2.338 μg mL^−1^	MTT	111.3 ± 0.3	−6.17 ± 0.41	Doxorubicin	24 h	Dose-dependent	Biotin	81	2016
		7.6 μg mL^−1^	MTT	209.4	−16.7	Paclitaxel	48 h	Dose-dependent	Pullulan	25	2016
		3.1 μg mL^−1^	MTT	218.2	−18.2	Combretastatin A4	48 h	Dose-dependent	Pullulan	25	2016
		∼2 μg mL^−1^	MTT	288.4 ± 1.11	−14.1	Epirubicin and tocotrienols	3 h	Dose-dependent	Asialofetuin	82	2014
	JHH-7	NA	MTT	175.25 ± 1.82	−19	Sorafenib	48 h	Dose-dependent	CXCR4 antagonist, AMD3100	83	2015
	SMMC-7721	NA	CCK-8	187.2 ± 10.6	+28.9 ± 0.3	Arsenic trioxide (As_2_O_3_)	24, 48 and 72 h	Time-dependent	Lactobionic acid-modified chitosan	84	2019
		NA	CCK-8	249.1 ± 9.1	−28.7 ± 1.1	As_2_O_3_	24, 48 and 72 h	Time-dependent	Lactose acid	85	2018
Ovarian	NCI/ADR-RES	0.044 ± 0.0026 μg mL^−1^	MTS	240 ± 1	−19 ± 5	Paclitaxel and tariquidar	96 h	Dose-dependent	Biotin	64	2009
	SKOV-3	NA	SRB	213.0 ± 3.5	−1.3 ± 3.8	Doxorubicin	24 h	Dose-dependent	HER2 antibody	22	2011
		0.062 μg mL^−1^	MTT	341.8 ± 86.8	−3.5 ± 5.9	Paclitaxel	3 h	Dose-dependent	TAT peptide (RKKRRQRRR)	86	2013
		0.073 ± 0.002 μg mL^−1^ (48 h), 0.027 ± 0.001 μg mL^−1^ (72 h)	MTT	184.6 ± 4.1	−28.6 ± 2.4	Docetaxel, silibinin and SPIONs	48 and 72 h	Time-dependent	LHRH decapeptide hormone	54	2016
		4.91 μg mL^−1^ (24 h), 1.42 μg mL^−1^ (48 h)	CCK-8	165.61 ± 13.36	+19.59 ± 1.74	Cisplatin	24 and 48 h	Time-dependent	Trastuzumab	87	2019
		NA	SRB	210 ± 7	−1.0 ± 0.5	Indocyanine green and doxorubicin	24 h	Dose-dependent	Anti-HER2	88	2014
	OCSCs	0.000754 μg mL^−1^	MTT	294.7	NA	Paclitaxel	24 h	Dose-dependent	FA	18	2017
Cervical	Luciferase-expressing HeLa	0.65 μg mL^−1^	CellTiter-Glo®	207 ± 4.461	+5.29 ± 1.5	Anti-luc siRNA	72 h	Dose-dependent	Lipid	89	2012
	HeLa	1.86 ± 0.17 μg mL^−1^	MTT	190.91 ± 0.19	−19.3 ± 0.59	Doxorubicin	24 h	Dose-dependent	Folate	90	2018
		0.80 ± 0.06 μg mL^−1^		190.91 ± 0.19	−19.3 ± 0.59		48 h	Time-dependent	Folate		2018
		1.66 ± 0.16 μg mL^−1^		194.97 ± 1.33	−15.4 ± 1.57		24 h	Dose-dependent	RGD peptide (Arg–Gly–Asp)		2018
		0.79 ± 0.13 μg mL^−1^		194.97 ± 1.33	−15.4 ± 1.57		48 h	Time-dependent	RGD peptide (Arg–Gly–Asp)		2018
		0.61 μg mL^−1^	MTT	151.7 ± 4.9	−1.00 ± 0.11	Paclitaxel	48 h	Dose-dependent	Glutamate	56	2016
	KB-3-1 and KB-V1	>9.95 μg mL^−1^	MTT	132.4 ± 1.5	−40.3 ± 6.1	Curcumin	48 h	Dose-dependent	Anti-P-glycoprotein	91	2012
	KB-V1	0.051 μg mL^−1^	MTT	165.4	−37.2	Curcumin	48 h	Dose-dependent	Mouse monoclonal anti-P-glycoprotein (P-gp) antibody (APgp; clone F4)	92	2014
	KB	NA	MTT	198 ± 12	−19.6 ± 1.5	Doxorubicin	48 h	Dose-dependent	FA	61	2016
		0.0057 μg mL^−1^	SRB	200–250	+9	Docetaxel and SPIONs	48 h	Dose-dependent	Folate–chitosan	19	2017
		2.7 μg mL^−1^	MTT	118 ± 3	−8.5 ± 2.4	Doxorubicin	10 h	Dose-dependent	FA	33	2015
	HCA-1	NA	MTT	175.25 ± 1.82	−19	Sorafenib	48 h	Dose-dependent	CXCR4 antagonist AMD3100	83	2015
Prostate	PC3	0.48 ± 0.087 μg mL^−1^	MTS	123 ± 2	−30.5 ± 1.4	Doxorubicin	72 h	Dose-dependent	Transferrin	93	2017
		NA	CellTiter-Glo®	207 ± 4.461	+5.29 ± 1.5	KIF11 siRNA	72 h	Dose-dependent	Lipid	89	2012
		0.093 ± 0.016 μg mL^−1^	WST-1	91.2 ± 8.1	−60.7 ± 1.0	Doxorubicin and irinotecan	240 h	Dose-dependent	Hyaluronic acid	60	2015
		0.04 μg mL^−1^	SRB	200–250	+9	Docetaxel and SPIONs	48 h	Dose-dependent	Folate–chitosan	19	2017
		0.01403 ± 0.00112 μg mL^−1^ (24 h), 0.00579 ± 0.00109 μg mL^−1^ (48 h), 0.00322 ± 0.00111 μg mL^−1^ (72 h)	MTT	146.9 ± 8.6	NA	Docetaxel and SPIONs	24, 48 and 72 h	Dose- and time-dependent	Single-chain prostate stem cell antigen antibodies	21	2011
	PC3M	NA	CCK-8	187.4 ± 32.7	NA	Docetaxel and SPIONs	24, 48 and 72 h	Dose- and time-dependent	Prostate stem cell antigen antibody	94	2012
	LNCaP	NA	CellTiter-Glo®	207 ± 4.461	+5.29 ± 1.5	KIF11 siRNA	72 h	Dose-dependent	Lipid	89	2012
	DU145	NA	CellTiter-Glo®	207 ± 4.461	+5.29 ± 1.5	KIF11 siRNA	72 h	Dose-dependent	Lipid	89	2012
		<80 μg mL^−1^	SRB	206.9	+21.7	Bicalutamide	120 h	Dose-dependent	FA	95	2015
Uterine	MES-SA/Dx5	NA	SRB	213.0 ± 3.5	−1.3 ± 3.8	Doxorubicin	24 h	Dose-dependent	HER2 antibody	22	2011
Pancreas	PANC-1	NA	Alamar blue	150	NA	Curcumin and SPIONs	48, 72, 96 h	Dose- and time-dependent	AS1411 Apt	96	2016
		NA	Alamar blue	150	NA	Curcumin and gemcitabine	120 h	Dose-dependent	AS1411 Apt	97	2013
	MIA PaCa-2	NA	Alamar blue	150	NA	Curcumin and SPIONs	48, 72, 96 h	Dose- and time-dependent	AS1411 Apt	96	2016
		NA	Alamar blue	150	NA	Curcumin and gemcitabine	120 h	Dose-dependent	AS1411 Apt	97	2013
	PK-1	NA	Alamar blue	150	NA	Curcumin and gemcitabine	120 h	Dose-dependent	AS1411 Apt	97	2013
	Normal hTERT-HPNE	NA	Presto blue and SRB	200 ± 10	−16 ± 1	Bortezomib	48 h	Dose-dependent	Human holo-transferrin	98	2014
	S2–013	0.0028 ± 0.00019 μg mL^−1^	Presto blue and SRB	200 ± 10	−16 ± 1	Bortezomib	48 h	Dose-dependent	Human holo-transferrin	98	2014
Skin	A375-luc	0.09 μg mL^−1^	MTS	200 ± 10	−16 ± 1	Cisplatin and rapamycin	24 h	Dose-dependent	Anisamide	99	2014
	B16F10	NA	Trypan blue	244.1 ± 4.3	−11.4 ± 2.3	Paclitaxel and combretastatin A4	24 h	Dose-dependent	cRGDfK peptide	100	2011
Umbilical vein endothelial	HUVECs	0.0396 μg mL^−1^	MTT	209.4	−16.7	Paclitaxel	48 h	Dose-dependent	Pullulan	25	2016
		0.0118 μg mL^−1^	MTT	218.2	−18.2	Combretastatin A4	48 h	Dose-dependent	Pullulan	25	2016
		NA	MTT	175.25 ± 1.82	−19	Sorafenib	48 h	Dose-dependent	CXCR4 antagonist, AMD3100	83	2015
		NA	Trypan blue	244.1 ± 4.3	−11.4 ± 2.3	Paclitaxel and combretastatin A4	24 h	Dose-dependent	cRGDfK peptide	100	2011
Oesophageal	OE21	NA	MTT	133	−4.7	Ruthenium-based DNA replication inhibitor and radiosensitizer (Ru(phen)_2_(tpphz)^2+^)	24 h	Dose-dependent	hEGF	27	2018
	OE33	NA	MTT	133	−4.7	Ruthenium-based DNA replication inhibitor and radiosensitizer (Ru(phen)_2_(tpphz)^2+^)	24 h	Dose-dependent	hEGF	27	2018
Bladder	UM-UC-3 and RT-4	0.509 μg mL^−1^	WST-1	151 ± 32	NA	Belinostat (NSC726630, PXD101)	72 h	Dose-dependent	Poly(guanidinium oxanorbornene)	101	2013
Head and neck	UMSCC 22A	NA	MTT	142	−14.6 ± 0.5	Doxorubicin	3 h	Dose-dependent	Anti-EGFR antibody (cetuximab)	29	2017
Kidney	COS-7	NA	MTT	118 ± 3	−8.5 ± 2.4	Doxorubicin	10 h	Dose-dependent	FA	33	2015

#### 2.1.1 Brain

Magnetic silica PLGA nanoparticles conjugated with transferrin showed the most potent cytotoxic effect against brain cancer cells (U-87) with an IC_50_ of 0.13 μg mL^−1^.^10^ This could be due to the dual drug delivery of doxorubicin and paclitaxel designed for the PLGA nanoparticles, compared to the single drug carriers listed in Table 1. The application of targeting ligands can improve the bioavailability of drug-loaded nanoparticles. In this study, transferrin was actively targeted to the overexpressed transferrin receptors in brain capillary endothelium and glioma cells. The IC_50_ was much lower when the treatment duration was prolonged from 48 h (1.03 μg mL^−1^) to 96 h (0.13 μg mL^−1^), showing the time-dependent effect of the treatment.

#### 2.1.2 Breast

Transferrin-conjugated lipid-coated PLGA nanoparticles carrying the aromatase inhibitor 7α-(4′-amino)phenylthio-1,4-androstadiene-3,17-dione (7α-APTADD) exhibited the greatest anti-proliferative effect against SKBR-3 breast cancer cells. The IC_50_ value of the nanoparticles was less than 0.00049 μg mL^−1^ for 24 h of treatment.^11^ These findings indicate that the inhibitory activity of nanoparticles has been improved in comparison with the non-targeted nanoparticles, accounting for the transferrin receptor-mediated endocytosis.

#### 2.1.3 Lung

Arginine–glycine–aspartic acid (RGD) peptide-modified and paclitaxel-loaded PLGA-chitosan nanoparticles (PTX-PLGA-CSNP-RGD) had the most potent cytotoxic effect against H1975 lung cancer cells with an IC_50_ of 0.0017 μg mL^−1^.^12^ The PTX-PLGA-CSNP-RGD nanoparticles showed enhanced uptake due to the nature of RGD peptide, which is highly targeted to the overexpressing integrin α_v_β_3_ receptor specifically found in lung cancer cells. In addition, less toxicity was received by the normal lung cells due to the weak expression of integrin α_v_β_3_ in normal lung cells.

#### 2.1.4 Colon

PLGA nanoparticles loaded with paclitaxel and conjugated with WGA wheat germ agglutinin (WNP) showed the most promising cytotoxic potency against colon cancer cells (HT-29) with an IC_50_ of 0.028 μg mL^−1^.^13^ WGA actively binds to the highly expressed *N*-acetyl-d-glucosamine-containing glycoprotein found in the membrane of colon cancer cells, thus increasing the cellular uptake of WNP in colon cancer cells. Since WGA tends to bind to the glycoprotein in colon cancer cells, WNP is more effective in delivering paclitaxel to colon cancer cells to enhance the bioavailability of paclitaxel compared to non-targeted nanoparticles.

#### 2.1.5 Stomach

Only one study about nanoparticles targeting stomach cancer cells was included for review after the databases were screened. PLGA nanoparticles modified with polyethylene glycol and conjugated with an engineered anti-human CD44v6 Fab (AbD15179) were developed to specifically target human CD44 isoforms containing exon v6 (CD44v6) present in stomach cancer cells. The PLGA nanoparticles were reported to exhibit anti-proliferative potency against GP202, MKN74-CD44v6+ and MKN74 stomach cancer cells. The cytotoxicity of the PLGA nanoparticles was 50 μg mL^−1^ and highly stable against fluid-mimicking gastrointestinal conditions.^14^ No IC_50_ data were reported in this study.

#### 2.1.6 Gastric

Dual-targeting hybrid nanoparticles made of PLGA and a lipoid shell prepared by conjugating the anti-HER2/neu peptides (AHNP) and *n*-hexadecylamine (HDA) to the carboxyl groups of hyaluronic acid (HA) were reported to deliver 7-ethyl-10-hydroxycamptothecin (SN38 agent) specifically to gastric cancer cells (HGC27 cells) with overexpression of (1) CD44 cluster determinant 44 and (2) HER2 (human epidermal growth factor receptor 2). An IC_50_ of 0.05 μg mL^−1^ was reported for the dual-targeting nanoparticles.^15^ Studies on the cytotoxicity mechanism have indicated that the enhanced cellular uptake of dual-targeting nanoparticles and suppression of CD44 and HER2 expression by HA and AHNP inhibit the growth of HGC27 cells.

#### 2.1.7 Liver

LFC131 peptide-conjugated PLGA nanoparticles composed of d-α-tocopheryl polyethylene glycol succinate (TPGS) moieties were prepared to deliver epirubicin and specifically bind with CXCR4-overexpressing human hepatic carcinoma cells (HepG2). TPGS is a vitamin E derivative and was used to stabilize the PLGA nanoparticles. It was also used as an inhibitor of P-glycoprotein in overcoming multi-drug resistance.^16^ LFC131 peptide-conjugated nanoparticles exhibited a threefold higher cellular uptake in HepG2 cells than non-targeted nanoparticles. CX-EPNP showed a promising anti-proliferative effect against HepG2 cells with an IC_50_ of 0.78 and 0.38 μg mL^−1^ for 24 and 48 h of treatment, respectively.^17^ Herein, LFC131 peptide-conjugated nanoparticles showed a time-dependent effect on the cytotoxicity studies.

#### 2.1.8 Ovarian

The potential use of PLGA nanoparticles as a paclitaxel carrier for ovarian cancer stem cells (OCSCs) was reported. PLGA nanoparticles loaded with paclitaxel were developed by an emulsion solvent evaporation method and then conjugated with a targeting ligand: folic acid (FA). Cytotoxicity results reveal that FA-conjugated nanoparticles had an IC_50_ of 0.00075 μg mL^−1^.^18^ Folate receptors (FR) are biomarkers that are over-expressed in human cancer cells, such as ovarian cancer cells. Therefore, FA was applied as the targeting ligand in the study to target paclitaxel to FR-positive OCSCs over the normal cells.

#### 2.1.9 Cervical

Magnetic PLGA nanoparticles with surface modified with folate–chitosan conjugate, which served as an anti-cancer and magnetic resonance imaging (MRI) contrast agent, were reported in one study. Docetaxel and super paramagnetic iron oxide nanoparticles (SPIONs) were loaded into the PLGA nanoparticles for delivery to folate-positive KB cancer cells. The folate–chitosan conjugate was prepared using the carbodiimide method and then used as a shell for the loaded PLGA nanoparticles to target the FR in KB cells. This specific targeting of FA in PLGA nanoparticles improved the cellular uptake by FR-positive KB cancer cells with an IC_50_ of 0.0057 μg mL^−1^.^19^

#### 2.1.10 Prostate

Theragnostic PLGA nanoparticles loaded with superparamagnetic iron oxide (SPIO) nanocrystals and docetaxel were prepared for both ultrasensitive MRI and cancer treatment. PLGA nanoparticles were formed by using a single emulsion evaporation method. The active targeting ligand single-chain prostate stem cell antigen antibodies (scAb_PSCA_) were conjugated to PLGA *via* a poly(ethylene glycol) linker. Overexpression of prostate stem cell antigen (PSCA), a prostate-specific glycosyl phosphatidylinositol-anchored glycoprotein found in prostate cancer PC3 cells, was the binding site for the scAb_PSCA_-conjugated nanoparticles.^20^ Targeted PLGA nanoparticles demonstrated improved cellular uptake and cytotoxicity in PC3 prostate cancer cells exhibited an IC_50_ of 0.01403 μg mL^−1^ (24 h), 0.00579 μg mL^−1^ (48 h) and 0.00322 μg mL^−1^ (72 h).^21^ Herein, the scAb_PSCA_-conjugated nanoparticles showed a time-dependent cytotoxicity against prostate cancer PC3 cells.

#### 2.1.11 Uterine

One study reported a comparison of the cytotoxicity and cellular uptake activity of targeted and non-targeted PLGA nanoparticles for delivering doxorubicin against multi-drug resistance in uterine (MES-SA/Dx5) cancer cells. HER2 antibody-conjugated nanoparticles and non-targeted nanoparticles showed higher cellular uptake of doxorubicin than free doxorubicin in MES-SA/Dx5 cancer cells. No significant difference was found regarding cytotoxicity in MES-SA/Dx5 cells for targeted and non-targeted PLGA nanoparticles. This was due to no HER2 receptor overexpression being observed in MES-SA/Dx5 cells. Higher cytotoxicity was observed for both targeted and non-targeted PLGA nanoparticles compared to free doxorubicin, showing suppression of the overexpression of P-glycoprotein in Dx5 cells. HER2 antibody-conjugated nanoparticles were able to overcome the multi-drug resistance (MDR) effect in Dx5 cells since cytotoxicity and cellular uptake results at 10 μM extracellular doxorubicin concentration were comparable.^22^

#### 2.1.12 Pancreas

Bortezomib, a proteasome inhibitor, was loaded into PLGA nanoparticles with poloxamer 407 as an emulsifier against S2-013 pancreatic cancer cells. Surface-modified of the PLGA nanoparticles with transferrin was done to achieve pancreatic cancer cell targeting. Cellular uptake studies have shown high uptake of the targeted PLGA nanoparticles by cancer cells for a sustained release of bortezomib from targeted PLGA nanoparticles. Targeted PLGA nanoparticles showed cytotoxic effects against pancreatic cancer cells with a GI_50_ of 0.0028 μg mL^−1^.^23^ Low toxicity to normal pancreatic cells demonstrated that the targeted PLGA nanoparticles enhanced the delivery of bortezomib to S2-013 pancreatic cancer cells.

#### 2.1.13 Skin

PLGA nanoparticles containing dioleoylphosphatidic acid (DOPA) coated cisplatin and rapamycin induced potent cytotoxic effects on A375-luc human melanoma cells with an IC_50_ of 0.09 μg mL^−1^.^24^ This was due to the synergistic effects of rapamycin and cisplatin towards A375-luc human melanoma cells. Rapamycin acts as a mammalian target of rapamycin inhibitors and a sensitizer. DOPA was coated onto cisplatin to achieve compatibility between PLGA and the dual drugs cisplatin and rapamycin. A high anti-proliferative effect was observed in PLGA nanoparticles conjugated with targeting ligand anisamide, which has a high affinity towards sigma receptor membrane-bound proteins that are overexpressed in A375-luc human melanoma cells.

#### 2.1.14 Umbilical vein endothelial

One study reported *in vitro* synergistic effects of paclitaxel or combretastatin A4-loaded charge reversible pullulan-conjugated PLGA nanoparticles formulated with poly(β-amino ester) for the treatment of human umbilical vein endothelial cells (HUVECs). This work revealed IC_50_s of less than 0.0396 and 0.0118 μg mL^−1^ for the respective paclitaxel and combretastatin A4-loaded nanoparticles.^25^ Pullulan-conjugated PLGA nanoparticles had high cytotoxicity activity in HUVECs due to the polysaccharide backbone of pullulan having a high affinity towards the asialoglycoprotein receptor (ASGPR) in HUVECs.^26^ The pH sensitivity of the PLGA nanoparticles was attributed to pullulan's cleavage of β-carboxylic amide bond towards changes in pH in the microenvironment of cells.

#### 2.1.15 Esophageal

Surface-modified PLGA nanoparticles with DTPA-hEGF allowed PLGA nanoparticles to attain radiolabeling and targeting towards the EGF receptor (EGFR). DTPA represents diethylenetriaminepentaacetic acid, while hEGF refers to human epidermal growth factor. Surface-modified PLGA nanoparticles were radiolabeled with ^111^In to achieve high affinity to EGFR-overexpressing esophageal cancer cells. The ^111^In radiolabeled PLGA nanoparticles induced radiotoxicity *via* cellular DNA damage. Ru1 or Ru(phen)_2_(tpphz)^2+^ (phen represents 1,10-phenanthroline, while tpphz represents tetrapyridophenazine) is a ruthenium-based DNA replication inhibitor and radiosensitizer. Ru1 was loaded into ^111^In radiolabeled PLGA nanoparticles for DNA damage enhancement. hEGF-PLGA-Ru1 nanoparticles showed remarkable cytotoxicity in EGFR-overexpressing OE21 cells due to the active targeting of the hEGF ligand. However, compared to OE21 cells, lower cytotoxicity was observed in EGFR-normal OE33 cells, with >70% proliferation.^27^ No IC_50_ data were reported for the *in vitro* cytotoxicity results of hEGF-PLGA-Ru1 nanoparticles.

#### 2.1.16 Bladder

PLGA nanoparticles were surface-modified with a novel cell-penetrating polymer – poly(guanidinium oxanorbornene) (PGON) – to improve tissue penetration tenfold in mouse bladder and human ureter. PGON is a synthetic polymer that mimics cell-penetrating peptides and possesses low toxicity to normal/cancer cells. PGON PLGA nanoparticles showed a significant enhancement in intracellular uptake of nanoparticles compared to unmodified nanoparticles. Belinostat, a histone deacetylase (HDAC) inhibitor, was loaded into the PGON PLGA nanoparticles to assess their biological activity. In comparison to uncapsulated belinostat, belinostat-loaded nanoparticles exhibited a significantly low IC_50_ (0.509 μg mL^−1^) in cultured bladder cancer cells (UM-UC-3 and RT-4) and sustained hyperacetylation.^28^

#### 2.1.17 Head and neck

A PLGA/polydopamine core/shell nanoparticle system was designed for light induced cancer thermochemotherapy. Overexpression of the epidermal growth factor receptor (EGFR) drove the high binding of anti-EGFR antibody-conjugated nanoparticles towards head and neck cancer cells. This enhanced cellular uptake of nanoparticles by head and neck cancer cells and induced the conversion of near-infrared light to heat, triggering drug release from the nanoparticles and cancer cell ablation due to the increased temperature. The study revealed that PLGA/polydopamine nanoparticles were effective in inhibiting cancer cells at 10 μM doxorubicin concentration when coupled with near-infrared (NIR) irradiation. A doxorubicin concentration of 5 μM or higher was required to achieve the NIR irradiation effect on PLGA/polydopamine nanoparticles in order to produce heat needed for cancer cell ablation and trigger drug release.^29^

#### 2.1.18 Kidney

Folate-targeted and reduction-triggered PLGA nanoparticles were prepared for targeted delivery of doxorubicin to the COS-7 kidney fibroblast-like cell line. Folate-targeted PLGA nanoparticles were prepared from a PLGA core containing a monolayer soybean lecithin and a reducible outer layer monomethoxy-poly(ethylene glycol)-*S-S*-hexadecyl (mPEG-*S-S*-C_16_). Disulfide bonds (-*S-S*-) are highly degradable in the reducing environment (high concentration of glutathione) of cancer cells and thus release drugs at the targeted site.^30–32^ FA was conjugated to the mPEG-*S-S*-C_16_ outer layer to achieve tumor targeting. No significant difference was observed in the cytotoxicity in FR-negative COS-7 cells for targeted and non-targeted PLGA nanoparticles. This shows that the cellular uptake of folate-targeted PLGA nanoparticles involved a FR-mediated endocytosis.^33^ The study showed a dose-dependent effect where the cytotoxic effect of the nanoparticles was directly proportional to the concentration of doxorubicin, which proves the non-cytotoxicity of the nanoparticles.

### 2.2 Size and cytotoxicity

**Fig. 3:**
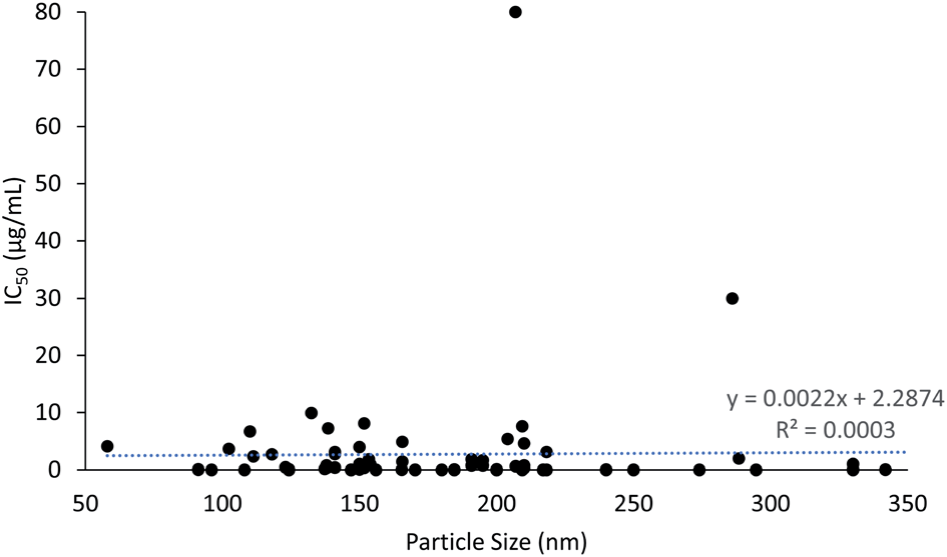
The correlation between particle size and cytotoxicity.

The cytotoxicity results show that PLGA nanoparticles induced an anti-proliferative effect as early as 3 h, taking 240 h at most. The particle size of PLGA nanoparticles ranged from 58 to 407 nm, with an average size of 189 nm. It was evident that particles smaller than 100 nm and larger than 300 nm were less commonly prepared, as a certain size is required to carry the load of the drugs and nor bigger size to promote the EPR effect in cancer cells. The IC_50_ of cytotoxicity of PLGA nanoparticles was plotted against particle size of the PLGA nanoparticles to assess the correlation between particle size and cytotoxicity (Fig. 3). It can be seen that as the particle size increased, the IC_50_ of cytotoxicity also increased, which indicates that smaller nanoparticles induced lower IC_50_ values and higher cytotoxicity potency. Although the application of corona materials such as hyaluronic acid increase the particle size, the efficacy of the encapsulated drugs to the desired site was enhanced. This is due to the cancer targeting properties provided by the binding of hyaluronic acid to the PLGA nanoparticles.^53,58,60,70,72^ Selective targeting enhances the cellular uptake of drugs in cancer cells and reduces the cytotoxic effect of drugs in normal cells. This further justifies that the ideal size and ability of PLGA nanoparticles to selectively deliver drugs to desired site are crucial in developing efficient and safe drug delivery systems. The IC_50_ values were mostly lower than 10 μg mL^−1^, but reached up to 80 μg mL^−1^ for FA-conjugated chitosan-functionalized PLGA nanoparticles (CPN) with particle size of 207 nm. This was due to the extremely low entrapment efficiency of bicalutamide in CPN (only about 1%).^95^

### 2.3 Zeta potential and cytotoxicity

**Fig. 4:**
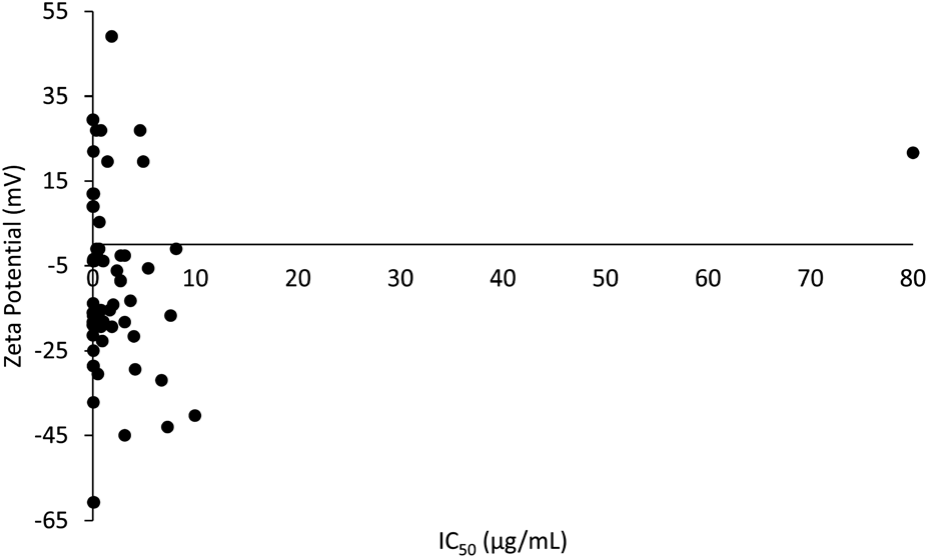
The correlation between zeta potential and cytotoxicity.

A plot of the zeta potential of the PLGA nanoparticles against their cytotoxicity IC_50_ values can be seen in Fig. 4. It is noticeable that PLGA nanoparticles were predominantly in negative charged because of its nature negatively charge of the carboxyl group end chain in PLGA. PLGA nanoparticles ranging from −13.2 to −19.3 mV showed the lowest IC_50_ value of 0.00031 μg mL^−1^ at −18.9 mV, with an average IC_50_ value of 1.15 μg mL^−1^ (*n* = 20) (ESI data†). Surface charge of the particle is defined by the absolute value of zeta potential. Zeta potential is a critical factor in designing a drug delivery system because it defines the stability of the nanoparticles. Interestingly, a former study reported that the cytotoxicity potency of PLGA nanoparticles is directly proportional to the absolute zeta potential.^102^ This is in agreement with a previous report that an absolute value of zeta potential 20 mV or much lower results in nanoparticles with adequate stability.^103^ The stability of nanoparticles is directly proportional to the absolute value of zeta potential. Since stronger repulsive forces were formed between the nanoparticles with high absolute values of zeta potential, stable nanoparticles with uniform size distributions were produced.^104^ PLGA is a hydrophobic polymer and can be stabilized by the hydrophilic corona materials such as chitosan.^19,47,84^ This is because of the absolute zeta potential of PLGA nanoparticles is increasing with the concentration of chitosan and indirectly resulted in increased stability of the PLGA nanoparticles. High stability in nanoparticles ensures that there is no early drug release along the route to the target site, which translates to a higher cytotoxic effect on the targeted site in cancer cells.

## 3. Discussion

This systematic review provides an overview of the application of different targeting ligands and active compounds/drugs used in PLGA nanoparticles to achieve active targeting for drug delivery to a particular cell line. This can give insight to researchers in regard to the designing of a potential drug delivery nanoparticle system for different types of cell lines. The potential of treating multiple cell lines using a single formulation makes the designation of a drug delivery system become more flexible in terms of its usage. For instance, a drug delivery nanoparticle system involving the encapsulation of paclitaxel and conjugation of transferrin as the targeting ligand can be used to treat several types of cancer cells, including breast and brain cancer cells. This can be explained by the fact that FR is a well-known biomarker that has a high affinity for FA in cancer cells due to its overexpression on several types of cancer cell lines, while paclitaxel has long been recognized as a mitotic inhibitor for treating various types of cancer cells. Both the drug and targeting ligand are suitable for treating breast and cervical cancers within a single formulation; this provides cost efficiency and compatibility to patients. The systematic review gives a summary of the IC_50_ of cytotoxicity of different types of cancer cells treated with various formulated PLGA nanoparticles, in which the overview of the cytotoxicity of PLGA nanoparticles is better understood. For instance, the exposure time and IC_50_ concentration for selected types of cells could serve as references for other researchers to use in planning and identifying their research interests and protocols, which would make these processes easier and save time.

The cellular uptake of nanoparticles with the presence of targeting ligands provides a specific binding to the target or cancer site without causing any or less harms to the healthy cells. This phenomenon is attracting more interest from researchers due to its safeness and less toxicity being imposed as a result of its specific controlled release of anti-cancer drugs. These statements are in line with the findings of previous studies that reported that the surface conjugation of a targeting ligand in nanoparticles more greatly enhances the cellular uptake of drugs and cytotoxicity potency compared to non-conjugated nanoparticles.^12,21,27^ For instance, the cell viability for RGD antibody-conjugated nanoparticles (PTX-PLGA-CSNP-RGD, 35.2%) was significantly lower (*p* < 0.01) than for non-conjugated nanoparticles (PTX-PLGA-CSNP, 45.7%).^12^ On top of this, 24 h treatment of A549 cells with FluTax-PLGA-CSNP-RGD, on the basis of the fluorescence intensity of fluorescent paclitaxel (FluTax), revealed higher uptake of 28 pg/1 × 10^5^ cells, compared to PTX-PLGA-CSNP by 28 pg/1 × 10^5^ cells. The reduced cell viability and higher fluorescence intensity of FluTax in RGD antibody-conjugated nanoparticles demonstrates the selective absorption of paclitaxel through integrin receptor-mediated endocytosis compared to non-targeted delivery at the same dose.

Identifying the zeta potential and particle size of PLGA nanoparticles is crucial because an ideal nanoparticle size can provide an EPR effect for cancer cells' uptake of drugs, while proper zeta potential can provide nanoparticles with high stability. From this systematic review, it is evident that nanoparticles <300 nm in size and ∼20 mV in absolute zeta potential are favorable. This is because a decent size (∼200 nm) is required for nanoparticles to load the drugs, while high zeta potential provides a uniform and narrow size distribution, as well as high stability.^105^ Since nanoparticles are highly stable, the leakage of drugs is negligible during transportation along the target site, which means greater cytotoxicity potency and cellular uptake of nanoparticles.

Challenges are always present in designing a drug delivery system. Although active targeting of nanoparticles reduces toxicity to normal cells, the conjugation of targeting ligands to the nanoparticles might increase the particle size. This is time-consuming, as optimization of the preparation step in making nanoparticles of an ideal size is required. In addition, targeting ligand conjugation might also reduce the release of drugs from the nanoparticles due to the probability that the ligand may serve as an external barrier when releasing the drug from the nanoparticles. The process of conjugating targeting ligands onto the surface of nanoparticles often involves a two-step reaction. This two-step reaction may cause leakage or early release of drugs from the nanoparticles due to the involvement of a reaction incubation period as well as sonication to minimize the agglomeration of nanoparticles.

The studies analyzed in this systematic review involved different factors, including length of exposure time, active compounds and targeting ligands used in assessing cytotoxicity assays of PLGA nanoparticles in a single cell line. Comparisons among the available studies for a particular cell line are difficult to make due to the variations in the factors involved. Hence, there is still a lack of ideally designed PLGA nanoparticle drug delivery systems. Moreover, some *in vitro* studies lack proper descriptions for data, such as IC_50_ data for cytotoxicity assessment. Additionally, different *in vitro* cytotoxicity assays have been used (*e.g.* MTT, MTS, SRB), which may have resulted in variations in cytotoxicity data.^106^ Hence, it is difficult to obtain completely accurate results from comparison studies of cytotoxicity for PLGA nanoparticles.

Currently, only 19 drug formulations based on PLGA have been approved by the FDA.^107^ This negligible number displays that the development of PLGA formulations is very challenging. Thus far, none of the 19 FDA approved PLGA formulations are based on PLGA nanoparticles as they are mainly composed of PLGA microparticles, solid implant and *in situ* gel. This lacking availability of PLGA nanoformulations further indicates that more evaluations on its efficacy and safety requirements are needed before getting approved for clinical use. Poor drug entrapment efficiency and drug release kinetics from PLGA nanoformulations are the main challenges faced in order to deliver drugs effectively to the target site. For instances, initial burst drug release is the typical issue of low efficacy in designing PLGA nanoformulations. Although PLGA nanoformulations were reported to be safe and selective targeting to the cancer cells in *in vitro* and animal studies, they are insufficient to prove that the same outcomes will be observed in human trials. Safety concerns of a drug delivery system are the top priority when it comes to establish their application.

Nevertheless, challenges still arise along with opportunities. Despite the great challenges, PLGA nanoparticles are still of interest due to its biocompatibility and particularly, an FDA approved material for drug delivery. Over the past few decades, a significant advancement in the development of PLGA nanoparticles for the application of drug delivery has led to the revolution of pharmaceutical industry. The uniqueness of PLGA nanoformulations, specifically their sustained and controlled drug release as well as cancer targeting properties provide the assurance of great potentials as promising nanocarriers. There are still great potentials in PLGA nanoformulations by modifying their physicochemical properties with better understanding of the physiology of the cancer cells and pharmacokinetics of the drug delivery system. In terms of biocompatibility and toxicity in biological systems, the physicochemical properties of PLGA nanoformulations play an important role.^108^ Particle size, surface charge and selective targeting properties of PLGA nanoformulations are critical benchmarks to consider when it comes to efficacy. The potential of these state-of-the-art innovations and strategies to develop PLGA nanoformulations are contributing to the advancement of cancer treatment. Nevertheless, intensive evaluations for pharmacokinetics, biodistribution and toxicity are essential before progressing PLGA nanoformulations into clinical studies.

## 4. Conclusions

In conclusion, PLGA nanoparticles with active targeting properties have higher cytotoxicity and cellular uptake of drugs compared to non-targeting nanoparticles, regardless of the types of cells studied. The size and zeta potential of PLGA nanoparticles play a crucial role in defining the resulting cytotoxicity. Therefore, in future studies, greater focus should be placed on assessing the pharmacodynamic, pharmacokinetic and toxicokinetic profiles of the drug delivery system using cancer-targeting PLGA nanoparticles. In addition, although the listed PLGA nanoparticles exhibited the potency of pharmacological actions based on the *in vitro* data, there was a lack of *in vivo* data for many of them. This raises concerns regarding efficacy and safety of usage in the application of PLGA nanoparticles in human trials. Therefore, more relevant *in vivo* data on the efficacy and toxicity of PLGA nanoparticles are desired before human clinical trials should be commenced, as a pharmaceutical formulation can only be considered successful when both safety of usage and efficacy are guaranteed.

## Author contributions

Hock Ing Chiu: methodology, validation, formal analysis, investigation, writing – original draft, writing – review & editing, visualization, project administration. Nozlena Abdul Samad: data curation, formal analysis, investigation Lizhen Fang: data curation, formal analysis, investigation. Vuanghao Lim: conceptualization, methodology, validation, formal analysis, writing – review & editing, supervision, project administration, funding acquisition.

## Conflicts of interest

There are no conflicts to declare.

## Supplementary Material

RA-011-D1RA00074H-s001
